# Anti-Obesity Effects of *Morus alba* L. and *Aronia melanocarpa* in a High-Fat Diet-Induced Obese C57BL/6J Mouse Model

**DOI:** 10.3390/foods10081914

**Published:** 2021-08-18

**Authors:** Na-Yeon Kim, Shalom Sara Thomas, Dae-Il Hwang, Ji-Hye Lee, Kyung-Ah Kim, Youn-Soo Cha

**Affiliations:** 1 Department of Food Science and Human Nutrition & Obesity Research Center, Jeonbuk National University, Jeonju 54896, Korea; nayeonkim986@gmail.com; 2 Department of Nutrition, University of Massachusetts Amherst, Amherst, MA 02204, USA; shalomsarathomas@gmail.com; 3 Institute of Jinan Red Ginseng, Jinan-gun 55442, Korea; dh5366@ijrg.re.kr (D.-I.H.); jh0108@ijrg.re.kr (J.-H.L.); 4 Department of Food and Nutrition, Chungnam National University, Daejeon 34134, Korea; kakim@cnu.ac.kr

**Keywords:** anti-obesity, *Morus alba*, *Aronia melanocarpa*, synergistic-effect

## Abstract

The present study investigated the synergic effect of extracts of *Morus alba* (MA) and *Aronia melanocarpa (Michx.)* (AR) against high-fat diet induced obesity. Four-week-old male C57BL/6J mice were randomly divided into five groups that were fed for 14 weeks with a normal diet (ND), high-fat diet (HD), HD with *M. alba* 400 mg/kg body weight (MA), HD with *A. melanocarpa* 400 mg/kg body weight (AR), or HD with a mixture (1:1, *v/v*) of *M. alba* and *A. melanocarpa* (400 mg/kg) (MA + AR). Treatment with MA, AR, and MA + AR for 14 weeks reduced high fat diet-induced weight gain and improved serum lipid levels, and histological analysis revealed that MA and AR treatment markedly decreased lipid accumulation in the liver and adipocyte size in epididymal fat. Furthermore, micro-CT images showed MA + AR significantly reduced abdominal fat volume. Expression levels of genes involved in lipid anabolism, such as SREBP-1c, PPAR-γ, CEBPα, FAS, and CD36 were decreased by MA + AR treatment whereas PPAR-α, ACOX1, and CPT-1a levels were increased by MA + AR treatment. Protein expression of p-AMPK and p-ACC were increased in the MA + AR group, indicating that MA + AR ameliorated obesity by upregulating AMPK signaling. Together, our findings indicate that MA and AR exert a synergistic effect against diet-induced obesity and are promising agents for managing obesity.

## 1. Introduction

Obesity is a clinical disease characterized by excessive body fat that poses various health problems. Obesity in Asian countries is diagnosed as a body mass index (BMI = body weight/height in meters^2^) greater than or equal to 25 kg/m^2^ based on World Health Organization criteria [1]. There is a close relationship between obesity and the incidence of metabolic syndrome, which comprises type 2 diabetes mellitus (T2DM), cardiovascular disease, hypertension, and dyslipidemia, all of which lower quality of life and are associated with increased mortality [2]. Current obesity treatments are lifestyle modification, therapy, pharmacotherapy, and bariatric surgery [3]. Research has focused on fat accumulation, absorption, adipocyte differentiation, lipogenesis, and lipolysis to develop functional foods or nutraceuticals that can ameliorate obesity [4,5].

*Morus alba* (MA), also known as silkworm mulberry, common mulberry or white mulberry is a plant in the family Moraceae. This species, which is native to India and northern China, has many beneficial health effects [6]. Compounds such as quercetin-3-(6-malonyl-glycoside), carotene, folic acid, folinic acid, vitamin D, succinic acid, and prenylflavanes have been isolated from the leaves of MA [7]. Extracts of MA leaves have also been showed to regulate a blood glucose levels related to T2DM [8]. Furthermore, Li et al. [9] reported that compounds isolated from leaves of MA inhibited lipid accumulation in 3T3-L1 adipocytes. MA has also been demonstrated to have anti-obesogenic effects by promoting adipocyte apoptosis and impeding pre-adipocyte differentiation and lipogenesis in high-fat diet (HD)-induced obese mice [10].

*Aronia melanocarpa (Michx.)* (AR)*,* widely known as black chokeberry, is native to eastern North America and eastern Canada and is a member of the Rosaceae family. It is easy to cultivate, and its fruits have numerous bioactive compounds. AR has been shown to have anti-T2DM effects [11], anti-fibrotic effects in the liver by inhibiting TGF-β signaling [12], anti-inflammatory effects [13], as well as hypoglycemic and hypolipidemic [14] properties. It also has neuroprotective effects and has been shown to improve cognitive and locomotor ability by increasing acetylcholinesterase activity [11], in addition to anti-hypertensive effects [15]. Bioactive compounds of AR include neochlorogenic and chlorogenic acid, anthocyanins, flavanols, flavonols [16]. Zhu et al. [17] reported that polyphenol-rich AR extract modulated the gut microbiota and improved lipid metabolism in an obese rat model.

Although a few studies have investigated the anti-obesogenic effects of MA or AR alone [9,10,17,18], no previous studies have examined their combined effect. Combined treatment with extracts from these two plant species could have a synergistic effect. Choi et al. showed that *Patrinia scabiosaefolia* and *Hippophae rhamnoides* extracts had a synergistic anti-obesity effect [19]. Similarly, crocin, chlorogenic acid, geniposide, and quercetin have been shown to reduce lipid accumulation in hepatic cells [20]. Given that MA and AR contain various bioactive compounds that may potentially interact a synergistically, our goal in this study was to determine if MA and AR interacted synergistically to ameliorate HD-induced obesity.

## 2. Materials and Methods

### 2.1. Preparation and HPLC Analysis of MA and AR

Powdered forms of MA leaves and AR fruits were obtained from Jinan-gun, Korea and were extracted twice, three hours from reflux extractor, each with 700 mL of 80% ethanol in 70 g powder. The extract was filtered with whatman N0.41 and concentrated with a rotary evaporator to remove the ethanol, freeze-dry, and manufactured in powder form. The compounds in the MA and AR was quantified by HPLC.

The compounds of MA were determined using Agilent 1200 infinity (Palo Alto, CA, USA) equipped with a FLD detector. A Capcell pak ADME 3UM column (4.6 × 150 mm) was used. Flow rate was set at 1.0 mL/min. The column temperature was continued at 35 °C. Injection volume was 10 μL. Excitation wavelength and emission wavelength were set to be 254 and 322 nm, respectively, for the analysis. A gradient elution using solvent A (0.1% formic acid in DW) and solvent B (acetonitrile) was used as follows: 0–10 min, 60% A and 40% B. The compounds of AR were determined using Agilent 1200 infinity (Palo Alto, CA, USA) equipped with a DAD detector. A Capcell pak ADME 3UM column (4.6 × 150 mm) was used. Flow rate was set at 1.0 mL/min. The column temperature was continued at 25 °C. Injection volume was 10 μL. Wavelength was set to be 310 nm. A gradient elution using solvent A (0.1% formic acid in DW) and solvent B (acetonitrile) was used as follows: 0–4 min, 90% A and 10% B; 4–15 min, 90% A and 10% B; 15–25 min, 70% A and 30% B; 25–30 min, 70% A and 30% B; 30–40 min, 90% A and 10% B. These were performed by the Institute of Jinan Red Ginseng (Jinan-gun, Korea).

### 2.2. Animal Experimental Protocol

All experimental protocols related to animals were endorsed by the Animal Ethics Committee of Jeonbuk National University (CBNU 2020–0123). Four-week-old C57BL/6J mice were purchased from Central Lab, Animal, Inc. (Seoul, Korea) and experimental diets obtained from DooYeol Biotech (Seoul, Korea). Upon arrival, mice were housed in cages under a 12 h light, 12 h dark cycle within the temperature (range of 25 ± 2 °C) and humidity of 50 ± 5%. After one week of adaptation, mice were divided into five groups each containing 10 mice: ND (normal diet control), HD (high-fat diet control), MA (high-fat diet supplemented with MA extract), AR (high-fat diet supplemented with AR extract), and MA + AR (high-fat diet supplemented with a mixture of MA + AR). The normal-diet (ND) group was fed a diet containing 10% kcal from fat (D12450B), and the high-fat diet groups were fed a diet containing 60% kcal from fat (D12492) for 14 weeks and the treatment with samples were started at the same time of experimental diet feeding. MA and AR groups received oral administration of individual extracts at 400 mg/kg body weight (bw), while the MA + AR group received extracts (400 mg/kg bw each) mixed at a 1:1 *v/v* ratio. Samples were dissolved in distilled water and orally administered 100 μL per mouse for 14 weeks, once a day. The control groups received oral administration of the same volume of distilled water.

Body weight was measured once a week. Feed intake was calculated thrice a week, by subtracting the remaining amount from the feed given per mice per cage. Mice were sacrificed after the experimental period after 12 h of overnight fasting and blood, liver, epididymal white adipose tissue were collected. Serum was collected by keeping the blood at room temperature for 1 h followed by centrifugation at 3000 rpm for 15 min at 4 °C. Epididymal fat and liver were dissected, weighed, and stored a −72 °C until further assay.

### 2.3. Analysis of Serum Biochemical Parameters

Serum triglyceride (TG), total cholesterol (TC), and high-density lipoprotein cholesterol (HDL-C) levels were determined by enzymatic methods using commercial kits (Asan Pharmaceutical Co., Seoul, Korea). Very-low-density lipoprotein cholesterol (VLDL-C) and low-density lipoprotein cholesterol (LDL-C) levels were calculated using Friedewald’s formula (Friedewald, Levy, and Fredrickson, 1972). Serum free fatty acids (FFAs) and glycerol concentrations were determined with assay kits (Abcam, Cambridge, UK) (FFAs, Cat# ab65341; glycerol, Cat# ab65337, ab202373). Serum insulin, adiponectin, and leptin concentrations were determined using ELISA kits according to the manufacturer’s protocols (insulin, Cat# 80-INSMS-E01, ALPCO Diagnostics, Salem, NH, USA; adiponectin and leptin, Cat# MRP300 and MOB00, respectively, R&D Systems, Minneapolis, MN, USA). Absorbance was measured using a microplate reader (MRX II, Dynex Technologies, Chantilly, VA, USA).

Hepatic lipids were extracted as described previously [21]. To measure hepatic FFAs and glycerol content, liver tissue was homogenized and analyzed using the same commercial kits used for serum.

### 2.4. Micro-Computed Tomography (CT)

Micro-CT was conducted using a high-resolution in vivo micro-CT system (Skyscan, Konitch, Belgium) at the Center for University-wide Research Facilities (CURF) of Jeonbuk National University to analyze visceral white adipose tissue (VAT) volume. Fat volume was calculated using Image J software.

### 2.5. Histological Analysis

Liver and adipocyte tissue were fixed with 10% formalin for 48 h and samples were processed further by the KP&T Company (Cheongju-si, Korea). Hematoxylin and eosin (H&E) staining and Oil Red O (ORO) staining were performed. Stained samples were analyzed by optical microscopy (DM2500, Leica, Germany) in the CURF of Jeonbuk National University. Image-J software (US National Institutes of Health, Bethesda, MD, USA) was used to determine adipocyte area.

### 2.6. Gene Expression Analysis

Expression levels of genes associated with lipid metabolism in the liver and adipose tissue were analyzed using real time-PCR (Applied Biosystems, Waltham, MA, USA). The following PCR cycle was performed: Stage 1; 95 °C for 10 min, Stage 2; (Step 1–95 °C for 15 s, Step 2–60 °C for 20 s, and Step 3–72 °C for 35 s) × 40 cycles. Briefly, mRNA from liver and adipose tissue was isolated using Trizol reagent (Life Technologies, Inc., Carlsbad, CA, USA). After quantification of RNA concentration, cDNA was synthesized using the PrimeScript RT Master Mix (Takara, Kyoto, Japan). Real time-PCR was conducted using SYBR green qPCR mix (Toyobo, Osaka, Japan). Primers used in RT-PCR are listed in Table 1.

### 2.7. Western Blot Analysis

Protein levels of p-AMPK, AMPK, ACC, p-ACC (Cell Signaling Technology, Danvers, MA, USA), and β-actin (Santa Cruz Biothechnology, Dallas, TX, USA) in the liver were determined by immunoblotting. Liver tissue (50–100 mg) was homogenized in RIPA lysis buffer (Pierce-Thermo Fisher Scientific Korea Ltd., Seoul, South Korea) containing 1% protease inhibitor and 1% phosphatase inhibitor cocktail (Merck, Seoul, South Korea), centrifuged (4 °C, 12,000× *g*, 15 min), and the supernatant was then collected. After measuring protein concentrations, equal concentration of samples was heated to 95 °C after mixing with 5X protein buffer. Then, proteins were electrophoresed on 8–10% SDS-polyacrylamide gels and transferred to polyvinylidene difluoride membranes (Bio-Rad Laboratories, Hercules, CA, USA) followed by blotting with antibodies.

### 2.8. Statistical Analysis

Data are expressed as means ± standard deviation of the mean (S.D). Statistical significance was verified using Duncan’s test with one-way ANOVA in SPSS version 17.0 (SPSS Inc., Chicago, IL, USA), and results were considered significant at *p* < 0.05. Values with different superscript letters (a, b, c) indicate statistically significant differences among groups.

## 3. Results

### 3.1. Compounds Present in MA and AR

**Figure 1:**
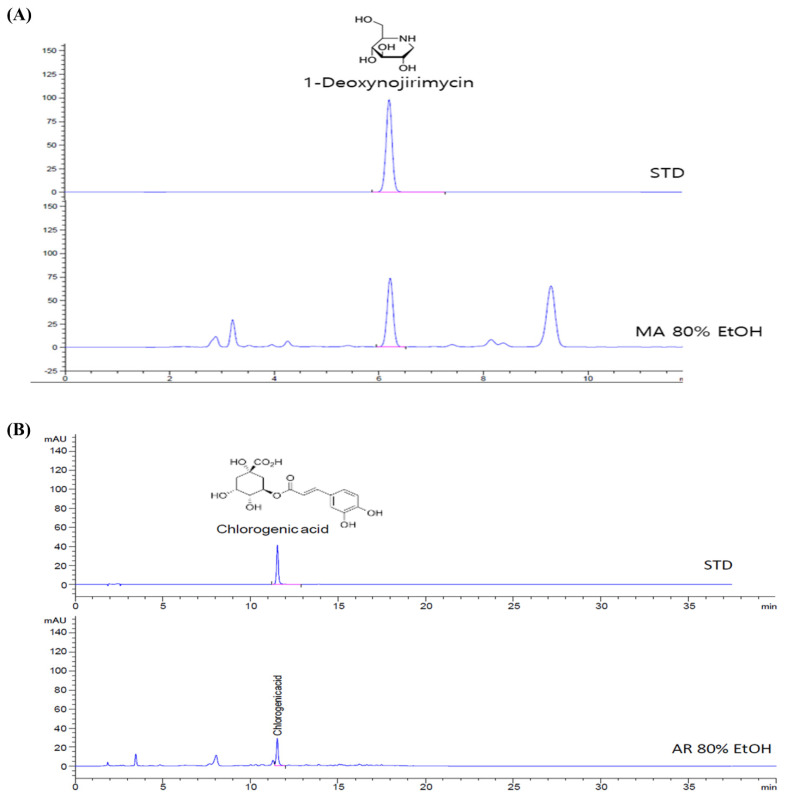
HPLC analysis of *Morus alba* (MA) leaf and *Aronia melanocarpa* (AR) fruit and chemical structure. (**A**) HPLC chromatogram of MA extract, (**B**) HPLC chromatogram of AR extract.

For HPLC analysis, the most commonly detected substance in the 80% ethanol extract of MA is 1-deoxynojirimycin. The amount of 1-deoxynojirimycin is 21.45 mg/g extract (Figure 1A). As shown in Figure 1B, chlorogenic acid was contained in the 80% ethanol extract of AR and its amount is 3.89 mg/g extract.

### 3.2. Effect of MA and AR Extracts on Body Weight, Tissue Weight, and Feed Intake

**Figure 2:**
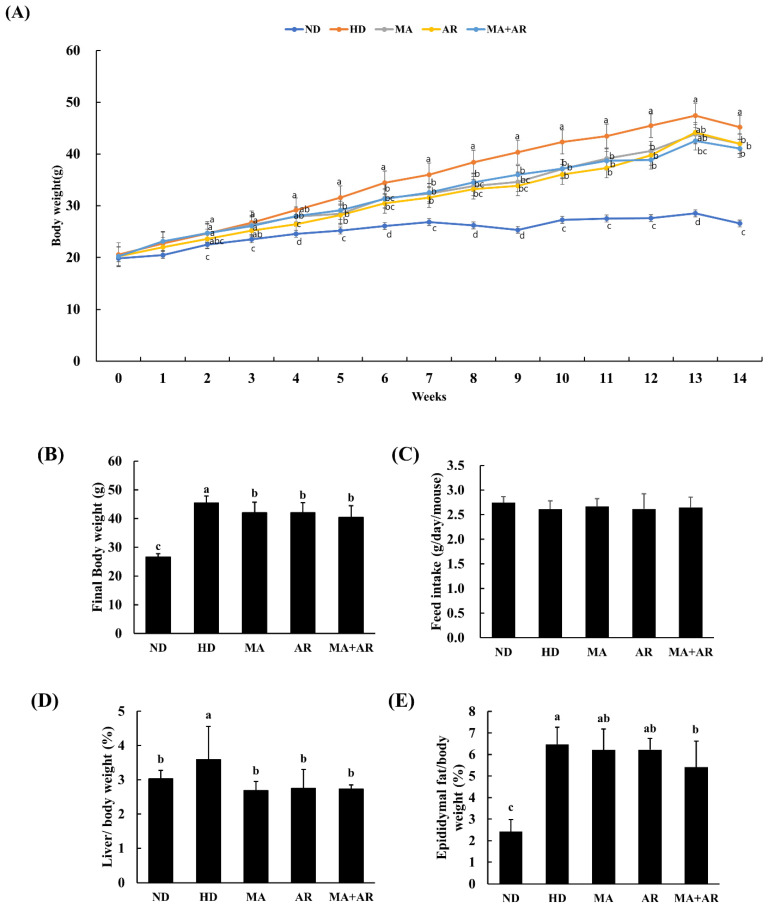
Effect of MA and AR on body weight (bw) and organ weight in experimental mice. *M. alba* extract (400 mg/kg bw), *A. melanocarpa* extract (400 mg/kg bw), or mixture of these two extracts was orally administered to experimental mice fed a high-fat diet for 14 weeks. (**A**) Growth curve during the experimental period, (**B**) final body weight, (**C**) feed intake for 14 weeks, (**D**) liver weight (g)/bw (g) %, (**E**) epididymal fat weight (g)/bw (g) %. ND, normal diet, HD, high-fat diet, MA, high-fat diet + *M. alba* 400 mg/kg bw, AR, high-fat diet + *A. melanocarpa* 400 mg/kg bw, MA + AR, high-fat diet + mixture of *M. alba* and *A. melanocarpa* (400 mg/kg bw). Results are expressed as means ± S.D (*n* = 10). Means with different superscript letters (a, b, c, d) are significantly different from each other by Duncan’s test of ANOVA at *p* < 0.05. “a” denotes the highest value and “d” represents the lowest value.

As shown in Figure 2A,B, body weight of all HD groups was significantly higher than that of the ND group. From week one of the experiment, the body weight of the ND group was significantly lower than that of the other groups. Treated groups showed a significant reduction in body weight compared to the HD group. Significant differences in body weight between the HD group and treated groups were observed starting at week 5. There were no significant differences in feed intake among these groups (Figure 2C). As shown in Figure 2D,E, the weights of the liver and epididymal fat to body weight were significantly greater in the HD group than the ND group. This HD-induced increase in liver weight was reversed in all the treatment groups. Epididymal fat weight was significantly decreased in all treated groups compared to the HD group, especially the MA + AR group.

### 3.3. Effect of MA and AR Treatment on Serum Lipid Profiles and Adipokine Levels

As shown in Table 2, HD group mice had significantly higher levels of all serum lipids profiles than the ND group except for FFAs. High-fat diet fed groups treated with MA or AR showed a significant reversal in levels of TC, TG, LDL-C, VLDL, FFAs, and glycerol compared to the HD group. Levels of serum adipokines differed significantly between the ND and HD groups. Treatment with MA or AR significantly increased adiponectin level, and decreased leptin and insulin levels. TC, TG, LDL-C, VLDL, FFAs, glycerol, adiponectin, leptin, and insulin levels differed significantly between the MA + AR group and the HD group.

### 3.4. Effect of MA and AR on Hepatic Lipids

Hepatic lipid profiles are shown in Table 3. HD mice had significantly higher levels of hepatic TC and TG than the ND group (*p* < 0.05). There was a significant difference in TC between the HD group and the groups treated with MA or AR alone. Hepatic TG were significantly reduced in all treatment groups compared to the HD group.

*M. alba* extract (400 mg/kg bw), *A. melanocarpa* extract (400 mg/kg bw), or mixture of these two extracts was orally administered to experimental mice fed a high-fat diet for 14 weeks. TC, total cholesterol, TG, triglycerides. ND, normal diet, HD, high-fat diet, MA, high-fat diet + *M. alba* 400 mg/kg bw, AR, high-fat diet + *A. melanocarpa* 400 mg/kg bw, MA + AR, high-fat diet + mixture of *M. alba* and *A. melanocarpa* (400 mg/kg bw). Results are expressed as means ± S.D (*n* = 10). Means with different superscript letters (a, b) are significantly different from each other by Duncan’s test of ANOVA at *p* < 0.05. “a” denotes the highest value and “b” represents the lowest value.

### 3.5. Changes in Visceral Fat Volume and Histopathology after MA and AR Treatment

**Figure 3:**
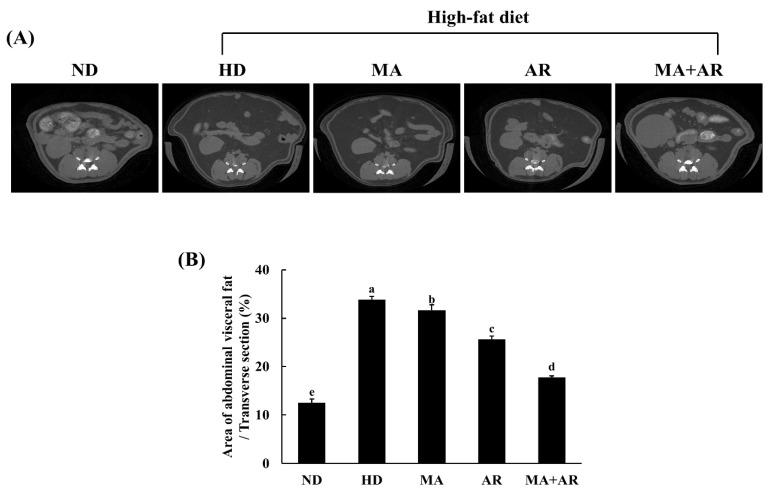
Micro-CT analysis of abdominal fat volume in the experimental mice. *M. alba* extract (400 mg/kg bw), *A. melanocarpa* extract (400 mg/kg bw), or mixture of these two extracts was orally administered to experimental mice fed a high-fat diet for 14 weeks. (**A**) Micro-CT image of abdominal mouse area, (**B**) percentage of visceral fat volume. ND, normal diet, HD, high-fat diet, MA, high-fat diet + *M. alba* 400 mg/kg bw, AR, high-fat diet + *A. melanocarpa* 400 mg/kg bw, MA + AR, high-fat diet + mixture of *M. alba* and *A. melanocarpa* (400 mg/kg bw). Results are expressed as means ± S.D (*n* = 10). Means with different superscript letters (a, b, c, d, e) are significantly different from each other by Duncan’s test of ANOVA at *p* < 0.05. “a” denotes the highest value and “e” represents the lowest value.

**Figure 4:**
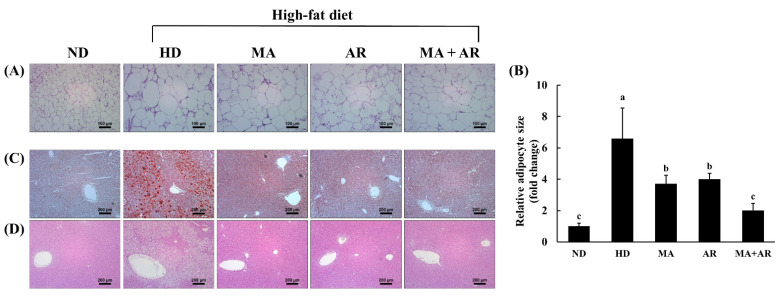
Histology of epididymal white adipose tissue (WAT) and liver in the experimental mice. (**A**) WAT stion (×100) by H&E staining, (**B**) adipocyte size of WAT, (**C**) liver stion (×200) by ORO staining, (**D**) liver stion (×200) by H&E staining. ND, normal diet, HD, high-fat diet, MA, high-fat diet + *M. alba* 400 mg/kg bw, AR, high-fat diet + *A. melanocarpa* 400 mg/kg bw, MA + AR, high-fat diet + mixture of *M. alba* and *A. melanocarpa* (400 mg/kg bw). Results are expressed as means ± S.D (*n* = 10). Means with different superscript letters (a, b, c) are significantly different from each other by Duncan’s test of ANOVA at *p* < 0.05. “a” denotes the highest value and “c” represents the lowest value.

Micro-CT was used to investigate changes in visceral fat volume after treatment. Visceral fat volume of the HD group was higher than that of the ND group (Figure 3). Compared with the HD group, the visceral fat volume of all treatment groups was reduced. The group treated with both MA and AR had a significantly reduced visceral fat volume than the group treated with each extract alone. As shown in Figure 4A,B, the size of epididymal white adipose tissue (WAT) in the HD group was significantly higher than that in the ND group based on H&E staining of epididymal WAT. Nevertheless, treatment with MA, AR, and MA + AR reduced adipocyte size. Oil Red O and H&E staining of liver tissue (Figure 4C,D) revealed that lipid droplets in the liver of HD group was more than in the ND group and HD-induced ectopic lipid accumulation was seemed to reversed by treatment with MA, AR, and MA + AR.

### 3.6. mRNA Expression of Genes in Liver and Adipose Tissue

**Figure 5:**
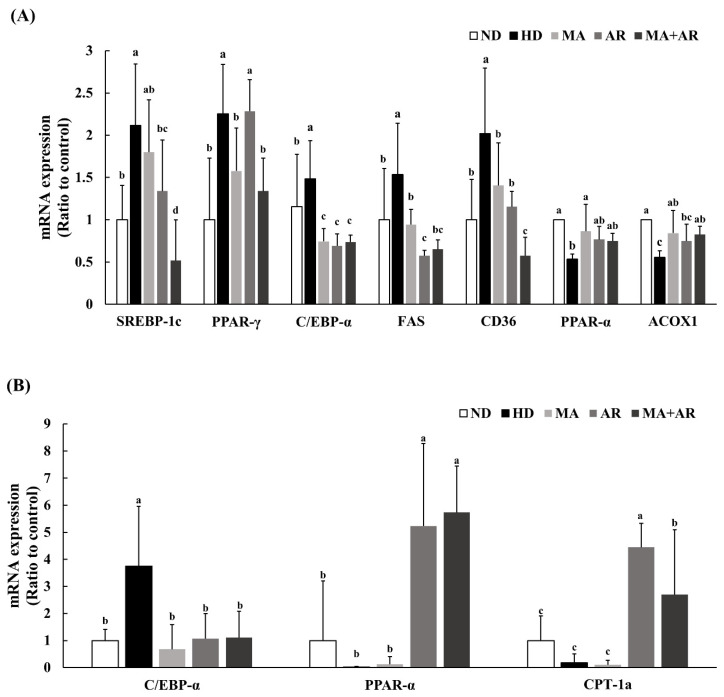
mRNA expression of genes related to lipid metabolism in liver and adipose tissue of the experimental mice. *M. alba* extract (400 mg/kg bw), *A. melanocarpa* extract (400 mg/kg bw), or mixture of these two extracts was orally administered to experimental mice fed a high-fat diet for 14 weeks. (**A**) Relative mRNA expression in liver, (**B**) relative mRNA expression in adipose tissue. ND, normal diet, HD, high-fat diet, MA, high-fat diet + *M. alba* 400 mg/kg bw, AR, high-fat diet + *A. melanocarpa* 400 mg/kg bw, MA + AR, high-fat diet + mixture of *M. alba* and *A. melanocarpa* (400 mg/kg bw). Results are expressed as means ± S.D (*n* = 10). Means with different superscript letters (a, b, c, d) are significantly different from each other by Duncan’s test of ANOVA at *p* < 0.05. “a” denotes the highest value and “d” represents the lowest value.

The mRNA expression of genes involved in lipid metabolism in the liver was analyzed using real time-PCR (Figure 5A). Expression of adipogenesis related genes, such as SREBP-1c, PPAR-γ, and C/EBP-α, was significantly higher in the HD group than the ND group. In the MA and AR groups, the expression of these genes was decreased compared to the HD group. There were significant differences in SREBP-1c, PPAR-γ, and C/EBPα expressions in the MA + AR group compared to HD group. The mRNA expression of genes related to fat accumulation, namely FAS and CD36, showed a similar pattern. Furthermore, mRNA expression of the lipolysis-related genes PPAR-α and ACOX1was increased in the MA, AR, and MA + AR groups compared with the HD group. As shown in Figure 5B, relative C/EBP-α expression in adipose tissue was significantly higher in the HD group than the other groups. Treatment with MA, AR, and MA + AR reduced expression of C/EBP-α. The expression of lipolysis-related genes such as PPAR-α and CPT-1a was significantly increased in the AR and MA + AR groups compared to the ND, HD, and MA groups.

### 3.7. Western Blot Analysis of Liver Tissue

**Figure 6:**
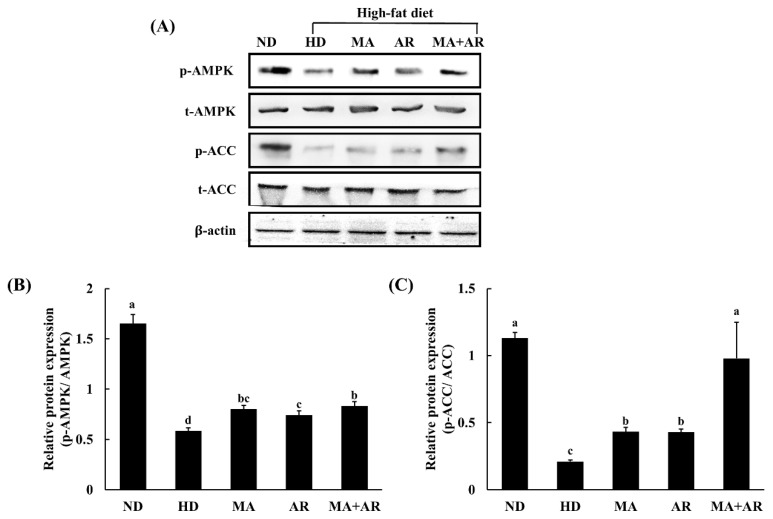
Proteins involved in lipid metabolism in the liver of the experimental mice. *M. alba* extract (400 mg/kg b.w), *A. melanocarpa* extract (400 mg/kg b.w) or mixture extract were orally administered to experimental mice fed a high-fat diet for 14 weeks. AMPK, adenosine monophosphate (AMP)-activated protein kinase, p-AMPK, phosphorylated AMPK, ACC, acetyl-CoA carboxylase, p-ACC, phosphorylated ACC. (**A**) Western blot, (**B**) relative expression of p-AMPK, and (**C**) relative expression of p-ACC. ND, normal diet, HD, high-fat diet, MA, high-fat diet + *M. alba* 400 mg/kg bw, AR, high-fat diet + *A. melanocarpa* 400 mg/kg bw, MA + AR, high-fat diet + mixture of *M. alba* and *A. melanocarpa* (400 mg/kg bw). Results are expressed as means ± S.D (*n* = 10). Means with different superscript letters (a, b, c, d) are significantly different from each other by Duncan’s test of ANOVA at *p* < 0.05. “a” denotes the highest value and “d” represents the lowest value.

As shown in Figure 6, levels of phosphorylated adenosine monophosphate (AMP)-activated protein kinase (p-AMPK) and phosphorylated-acetyl-CoA carboxylase (p-ACC) were significantly higher in all treatment groups compared with the HD group. The MA + AR group showed a marked difference in expression of p-ACC compared to the other treatment groups. These results suggest that the combination of MA and AR has an anti-obesity effect by targeting the AMPK pathway and promoting the phosphorylation of ACC, thereby suppressing lipogenesis.

## 4. Discussion

The prevalence of obesity has increased during the past 35 years to the extent that more than one third of the world’s population are overweight or obese [22]. Obesity is diagnosed based on BMI in addition to changes in FFAs, and insulin levels, as well as vascular muscle tone and glucose levels. Changes in physiology are dependent on the regional distribution of body fat, and can result in insulin resistance, T2DM, cardiovascular diseases, hypertension, and chronic respiratory failure [23]. Experts advise reducing body weight and changing lifestyle by eating balanced meals and increasing energy expenditure to remain healthy. Therapeutic drug intervention can help in achieving a normal body weight. However, several anti-obesity drugs have been reported to have adverse reactions, such as nausea, and cardiovascular and pulmonary hypertension [24]. Nutraceutical and herbal medicines from natural compounds are, therefore, being actively researched [25].

In the present study, we investigated the anti-obesity effects of the combination of MA and AR in a HD-induced obese mouse model. MA, AR, or a mixture of these two extracts was administered orally to mice for 14 weeks. Treatment with MA and AR, reversed obesity-induced changes in blood lipids, serum hormones, liver weight, and epididymal fat weight, as much as individual treatment with these extracts. Moreover, the expression of lipolysis-related genes in liver and adipose tissue was significantly increased by MA + AR treatment.

Continuous consumption of a high-fat diet leads to a continual increase in body weight and fat mass [26]. The increased body weight of all HD-fed groups compared to the ND group indicated successful induction of obesity. A high-fat diet induces accumulation of fat in organs, such as the heart, liver, intestine, and muscle [27,28], contributing to the increase in body weight. All treatment group showed less ectopic lipid accumulation in the liver than the HD group, as evidenced by a reduction in the number of globules in ORO-stained and H&E-stained liver tissue stions. Furthermore, abdominal fat volume was also reduced by MA, AR, and MA + AR treatment. Together, these changes explain the reduction in body weight in the treated groups. A previous study reported that MA and AR reduced tissue and body weight, consistent with our findings [10,18].

Persistent dyslipidemia increases the risk of T2DM and vascular diseases, such as stroke, hypertension, and coronary artery disease (CAD) [29]. In the present study, MA and AR treatment decreased TC, TG, LDL-C, and VLDL levels, consistent with a previous report [18,30]. FFAs are present in the blood in obesity, and increase insulin resistance, glucose production, inhibit movement of glucose into muscle cells, and downregulate the expression of the insulin receptor. FFAs are, therefore, associated with insulin resistance and T2DM [31,32]. All treatment group showed significantly reduced serum FFAs and glycerol levels compared to the HD group, indicating that treatment with MA, AR, and the combination of these two extracts improved obesity-induced T2DM. Leptin is a hormone produced and sreted by adipocytes in direct relation to the volume of body adipose tissue. Leptin regulates appetite, hunger, and satiety through the central nervous system [33]. Visceral obesity increases leptin concentration and causes leptin resistance. In contrast, adiponectin level is inversely proportional to body fat mass. Adiponectin deficiency causes metabolic syndrome, insulin resistance, and cardiovascular disease [34]. Previous studies showed that MA and AR decreased leptin and increased adiponectin levels in serum [18,35]. In our study, leptin level was significantly reduced in the MA + AR group compared to the HD group, while MA and AR groups showed a tendency towards reduced leptin concentrations compared to the HD group. There were significant differences in adiponectin levels between the MA, AR, and MA + AR groups compared with the HD group. A decreased adiponectin level increases glycemia by increasing gluconeogenesis and reducing glucose uptake and it is associated with insulin resistance and, therefore, T2DM. In addition, because insulin promotes fat synthesis in the liver, high levels of insulin are produced in obese-patients [29]. Insulin levels and body weight were significantly reduced in all treatment groups compared to the HD group. These results indicate that MA and AR improve insulin resistance [36,37]. There are several prior studies on the anti-obesity effect of 1-deoxynojirimycin detected in MA. The 1-deoxynojirimycin is potential α-glucosidase inhibitor, reduces the adipocyte size, regulates lipid parameters in the liver and blood, and activate β-oxidation. A factor of activation β-oxidation is adiponectin [38]. Overall, MA and AR ameliorated the symptoms and complications of obesity in our mouse model.

Hormones and adipokines produced by adipose tissue play a central role in obesity. PPAR-γ and C/EBP-α play key roles in pre-adipocyte differentiation and adipogenesis [39]. Expression of these genes is regulated by the transcription factor SREBP-1c that is expressed in adipose tissue and liver, and the proteins encoded by these genes synthesize TG from the surplus energy sources ingested. SREBP-1c is responsible for the synthesis of fatty acids and TG by controlling the expression of FAS [40,41,42]. FAS is key enzyme in de novo lipogenesis that produces first fatty acids, increases insulin resistance, and converts malonyl-CoA to palmitate [43]. CD36 is a membrane-bound protein expressed on monocytes, macrophages, thrombocytes, myocytes, and adipocytes that regulates the uptake of fatty acids across the membrane as a transporter [44]. In our study, the mRNA expression of SREBP-1c, PPAR-γ, C/EBP-α, FAS, and CD36 in liver tissue showed decreased tendency in all treatment groups. In the MA + AR group, a significant decrease in expression of all these genes was found compared to the HD group. In the adipose tissue, the expression of C/EBP-α, an important transcription factor involved in adipogenesis, was reduced in all the treated groups compared to the HD group indicating that MA, AR, and MA + AR reduced adipogenesis in the obese mice model. Furthermore, the expression of PPAR-α and CPT-1a were increased in AR and MA + AR treated groups. Activation of PPAR-α in adipose tissue accounts for its reduced adipocyte size and increased insulin sensitivity [45]. In addition, the transcription factor, PPAR-α has been suggested to be a therapeutic target for obesity [46] as targeting of this gene can improve serum lipid profiles, lipoprotein metabolism, and fatty acid β-oxidation [47]. ACOX1 is the first enzyme in the fatty acid β-oxidation pathway and has a positive relationship with PPAR-α [48]. CPT-1a catalyzes the conversion of fatty acyl-CoA to fatty acyl carnitine. This is regulated by PPAR-α and is the first step in mitochondrial oxidation [49]. The MA group had increased expression of PPAR-α in the liver compared to the HD group, and expression of PPAR-α was increased in the MA + AR group, albeit not significantly. However, ACOX expression in the liver was significantly higher in the MA + AR group than the HD group. This suggests a synergistic interaction between MA and AR that promote β-oxidation of fatty acids. Altogether, the results of the present study indicated that MA and AR in synergy could improve obesity by decreasing lipogenesis and increasing β-oxidation in the body. A previous study reported that MA ethanol extract had an anti-adipogenic effect by decreasing adipogenic genes expression in differentiated adipocytes [10]. AR has been shown to have an anti-obesity effect by downregulating the expression of SREBP-1c, PPAR-γ, and C/EBP-α [18]. The results of our study showed a similar pattern.

Activation of AMPK has been shown to reduce body weight gain HD-induced obese mice [50]. AMPK is involved in lipid metabolism by inhibiting acetyl-CoA carboxylase (ACC). AMPK induces phosphorylation of ACC, thus inhibits lipogenesis [51]. In addition, AMPK also upregulates β-oxidation by increasing the expression of PPARα. A previous study reported that chlorogenic acid-rich berry consumption improves insulin sensitivity by upregulating AMPK, PPAR-α, and ACOX [52]. The results of the present study were also in agreement with these results.

**Figure 7:**
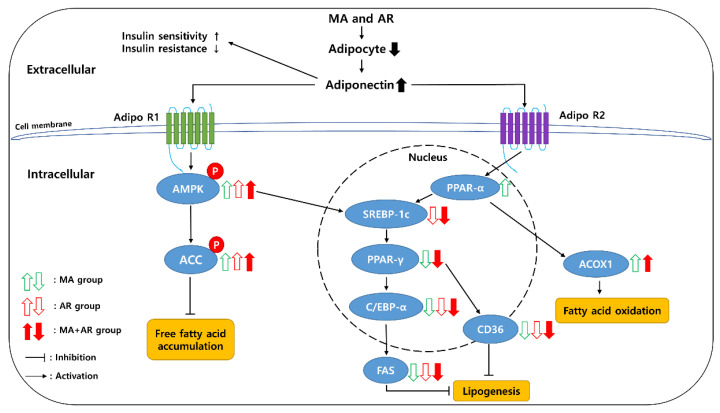
Schematic mechanistic diagram showing anti-obesity effect of MA and AR.

The activation of AMPK was noticeably increased in MA + AR group indicating that action of MA + AR against HD-induced obesity is through down regulation of lipogenesis and upregulation of β-oxidation. Figure 7 shows the anti-obesity mechanism of MA and AR.

Clinical trials of MA extracts have been conducted in reducing cholesterol levels and blood glucose on the hypolipidemic and hypoglycemic effects [53]. AR has also been found in clinical trials to cholesterol and blood pressure [54]. However, clinical trials that studied both MA and AR together are insufficient. Clinical trials are needed to evaluate synergistic effective and safe doses of these extracts in humans.

## 5. Conclusions

In summary, we observed that MA and AR interacted synergistically to ameliorate the negative effects of obesity compared to each of these extracts individually in C57BL/6 mice. MA and AR extracts significantly reduced the HD-induced increase in body weight. Serum and liver biochemical parameters, such as TG, LDL-C, VLDL-C, total-cholesterol, FFAs, glycerol, and adiponectin improved upon MA and AR treatment. These changes were associated with changes in the expression of transcription factors and coactivators related to adipogenesis and lipolysis. Furthermore, both MA and AR stimulated AMPK and ACC phosphorylation in liver tissue in a synergistic manner. Taken together, these results demonstrated that MA and AR had a synergistic anti-obesity effect.

## Figures and Tables

**Table 1:** List of primers used for PCR.

Gene Name ^1^	Primers	Sequence (5′→ 3′)
SREBP-1c	Forward	ACGGAGCCATGGATTGCACA
	Reverse	AAGGGTGCAGGTGTCACCTT
PPAR-γ	Forward	GCCCACCAACTTCGGAATC
	Reverse	TGCGAGTGGTCTTCCATCAC
C/EBP-α	Forward	GTGTGCACGTCTATGCTAAACCA
	Reverse	GCCGTTAGTGAAGAGTCTCAGTTTG
FAS	Forward	GAAGTGTCTGGACTGTGTCATTTTTAC
	Reverse	TTAATTGTGGGATCAGGAGAGCAT
CD36	Forward	GCTTGCAACTGTCAGCACAT
	Reverse	GCCTTGCTGTAGCCAAGAAC
PPAR-α	Forward	AGGCTGTAAGGGCTTCTTTCG
	Reverse	GGCATTTGTTCCGGTTCTTC
ACOX1	Forward	GTATAAACTCTTCCCGCTCCTG
	Reverse	CCAGGTAGTAAAAGCCTTCAGC
CPT-1a	Forward	TGGCATCATCACTGGTGTGTT
	Reverse	GTCTAGGGTCCGATTGATCTTTG

^1^ SREBP-1c, sterol regulatory-element binding proteins I; PPAR-γ, peroxisome proliferator-activated receptor gamma; C/EBP-α, CCAAT/enhancer-binding protein alpha; FAS, fatty acid synthase; CD36, cluster of differentiation 36; PPAR-α, peroxisome proliferator-activated receptor alpha; ACOX1, acyl-coenzyme A oxidase 1; CPT-1a, carnitine palmitoyltransferase I alpha.

**Table 2:** The effect of MA, AR on serum profiles in the experimental mice.

Parameters	ND	HD	MA	AR	MA + AR
TC (mg/dL)	111.2 ± 21.2 ^c^	192.4 ± 11.4 ^a^	184.7 ± 12.5 ^a^	178.4 ± 17.9 ^ab^	163.3 ± 22.2 ^b^
TG (mg/dL)	88.0 ± 13.6 ^b^	120.3 ± 20.7 ^a^	102.1 ± 13.5 ^b^	99.5 ± 10.6 ^b^	96.1 ± 11.6 ^b^
HDL-C (mg/dL)	47.5 ± 4.7 ^b^	56.8 ± 4.4 ^a^	60.5 ± 3.6 ^a^	59.3 ± 2.2 ^a^	60.4 ± 6.4 ^a^
LDL-C (mg/dL)	48.8 ± 20.7 ^c^	112.4 ± 14.6 ^a^	106.5 ± 10.3 ^ab^	99.4 ± 15.7 ^ab^	91.7 ± 13.6 ^b^
VLDL (mg/dL)	17.6 ± 2.7 ^b^	24.1 ± 4.1 ^a^	20.4 ± 2.7 ^b^	20.7 ± 3.1 ^b^	19.2 ± 2.3 ^b^
Free fatty acid (nmol/μL)	0.90 ± 0.01 ^ab^	0.99 ± 0.11 ^a^	0.71 ± 0.14 ^b^	0.75 ± 0.22 ^b^	0.74 ± 0.14 ^b^
Glycerol (nmol/μL)	0.22 ± 0.03 ^b^	0.28 ±0.03 ^a^	0.24 ± 20.02 ^b^	0.24 ± 0.03 ^b^	0.22 ± 0.03 ^b^
Adiponectin (ng/mL)	10.32 ± 0.30 ^a^	9.54 ±0.38 ^b^	11.05 ± 0.39 ^a^	10.51 ± 0.97 ^a^	10.50 ± 0.88 ^a^
Leptin (ng/mL)	4.15 ± 1.81 ^c^	75.24 ± 13.84 ^a^	61.19 ± 15.78 ^ab^	59.93 ± 9.51 ^ab^	56.08 ± 13.7 ^b^
Insulin (ng/mL)	0.06 ± 0.03 ^c^	1.44 ± 0.56 ^a^	0.66 ± 0.38 ^b^	0.71 ± 0.46 ^b^	0.98 ± 0.63 ^b^

*M. alba* extract (400 mg/kg bw), *A. melanocarpa* extract (400 mg/kg bw), or mixture of these two extracts was orally administered to experimental mice fed a high-fat diet for 14 weeks. TC, total cholesterol, TG, triglycerides, HDL-C, high-density lipoprotein cholesterol, LDL-C, low-density lipoprotein-cholesterol, VLDL, very low density-lipoprotein cholesterol. ND, normal diet, HD, high-fat diet, MA, high-fat diet + *M. alba* 400 mg/kg bw, AR, high-fat diet + *A. melanocarpa* 400 mg/kg bw, MA + AR, high-fat diet + mixture of *M. alba* and *A. melanocarpa* (400 mg/kg bw). Results are expressed as means ± S.D (*n* = 10). Means with different superscript letters (a, b, c) are significantly different from each other by Duncan’s test of ANOVA at *p* < 0.05. “a” denotes the highest value and “c” represents the lowest value.

**Table 3:** The effect of MA, AR on hepatic lipids in the experimental mice.

Parameters	ND	HD	MA	AR	MA + AR
TC (mg/g tissue)	17.3 ± 1.6 ^b^	23.7 ± 4.0 ^a^	18.8 ± 3.0 ^b^	21.0 ± 3.6 ^b^	21.9 ± 3.9 ^ab^
TG (mg/g tissue)	50.3 ± 7.7 ^b^	66.1 ± 8.0 ^a^	55.0± 8.7 ^b^	49.9 ± 10.2 ^b^	55.9 ± 8.6 ^b^

## Data Availability

The data presented in this study are available upon request to the corresponding author.
